# Soft tissue sarcoma affecting the right shoulder of a man with paraplegia from a remote traumatic spinal cord injury: a case report

**DOI:** 10.1038/s41394-018-0081-8

**Published:** 2018-06-26

**Authors:** Colin K. Franz, Kyriakos Dalamagkas, Lewis A. Jordan, Gayle R. Spill

**Affiliations:** 1 Biologics Laboratory, Shirley Ryan AbilityLab (formerly Rehabilitation Institute of Chicago), Chicago, IL USA; 2 Department of Physical Medicine and Rehabilitation, Northwestern University Feinberg School of Medicine, Chicago, IL USA; 3 The Ken & Ruth Davee Department of Neurology, Division of Neuromuscular Medicine, Northwestern University Feinberg School of Medicine, Chicago, IL USA; 4 The Institute for Rehabilitation and Research, Memorial Hermann Texas Medical Center, Houston, TX USA; 5 Cancer Rehabilitation Program, Shirley Ryan AbilityLab (formerly Rehabilitation Institute of Chicago), Chicago, IL USA

## Abstract

**Introduction:**

People with spinal cord injury (SCI) are getting older due to a combination of increased life expectancy and older age at the time of injury. This trend makes it more likely for these patients to have other chronic health conditions including cancer. Inevitably relatively rare cancers such as soft tissue sarcomas (STS), which are more common with advancing age, will occur in some SCI patients. The present case represents the first report of a limb STS in a patient with chronic paraplegia from a traumatic SCI.

**Case presentation:**

We report a case of a 50-year-old right handed male with a T6 chronic, complete SCI (American Spinal Injury Association Impairment Scale A) who presented with a large mass involving his right shoulder musculature that was determined to be a high grade spindle cell sarcoma. The patient was followed closely by Physiatry over an approximately 6-month time course including prior to his tumor diagnosis, during the pre-radiation and pre-surgical planning phase, and then post-operatively for his acute inpatient rehabilitation. He was successfully discharged home to live alone in his accessible apartment complex.

**Discussion:**

This case is the first ever reported case of a person living with a traumatic SCI who subsequently developed a limb STS. In addition to its novelty, this case illustrates how health conditions such as rare cancers are presenting more often as the chronic SCI population is getting older, which creates both unique diagnostic and management challenges for cancer rehabilitation specialists.

## Introduction

Soft tissue sarcomas (STSs) are a rare type of tumors that account only for 1% of all tumors [1]. According to the American Cancer Society (ACS) 2018 statistics, there were 13,040 new cases of STSs and 5,150 reported STSs-related deaths during the year [2]. Identified risk factors that increase the risk for STSs are: previous exposure to radiation, certain family cancer syndromes, a damaged lymph node system, such as previous lymphedema, immunodeficiency accompanied by viral infections and different types of chemicals [3]. Trauma in the affected area is also suspected as a cause of STSs. Clinical signs and symptoms are usually non-specific. The most common clinical presentation is a patient with a gradually enlarging mass in the affected area without pain. There are at least 50 subtypes of STSs according to WHO and staging is based on both clinical and pathological features [4–6]. The five-year survival rates are 86, 72, 52, and 15% for stages I, II, III and IV, respectively [7]. Surgery is the mainstay therapy in STSs and radiation therapy has an adjuvant and neo-adjuvant role in the treatment. The recurrence rate of STSs of the limbs is 5–10% and 2/3 of the recurrences or metastasis occur in the first 2 years after the resection [7].

Like anyone else, people with traumatic spinal cord injury (SCI) have a significant lifetime risk of developing cancer. The average age of people living with traumatic SCI is trending higher [8, 9], which puts this population at greater risk for cancer and other chronic health conditions. Naturally rare diseases such as STSs are expected to be as uncommon in the SCI population as they are in the general population. However, given that surgical resection is the treatment of choice for limb-STS, this case highlights the unique management challenges for the physiatrist and surgeon when this aggressive disease affects the upper extremity in a patient with paraplegia after SCI.

## Case presentation

**Fig. 1:**
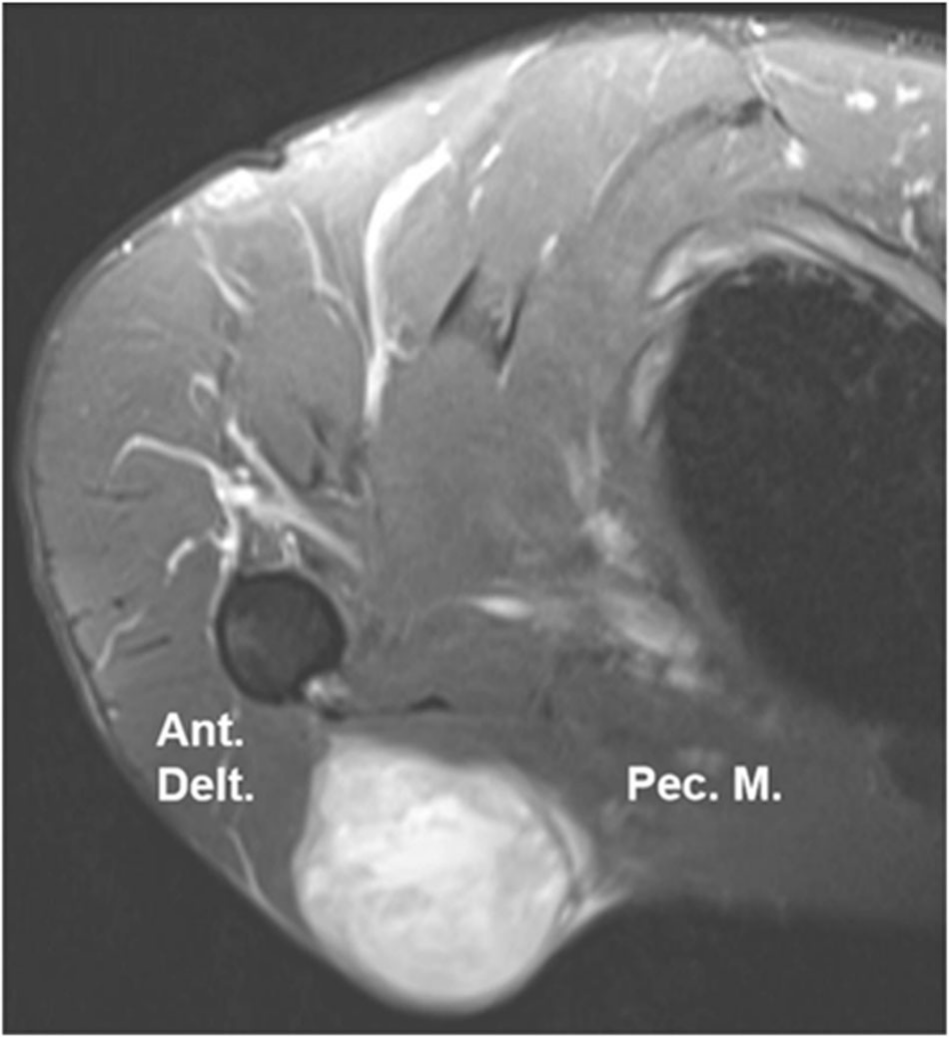
T2 MRI sequence in the axial plane that demonstrates the involvement of anterior deltoid (Ant. Delt.) and pectoralis major (Pec. M.) muscles by this patient’s soft tissue sarcoma pre-operatively

This is a 50-year-old right-handed male, with 33-year history of T6 AIS A SCI from a gunshot wound complicated by chronic pain, left hip and knee heterotophic ossification, and a chronic dislocation of his right hip, who initially presented to the emergency room with a right shoulder mass in September 2014. While he initially noticed the mass about 2 months earlier, he presented for evaluation now because of acute onset of pain, weakness and paresthesias in the right arm. He was admitted to the general medicine service for pain management and underwent an initial work up for his right shoulder mass, including advanced imaging and a core biopsy. Physiatry was consulted due to his functional deterioration that precluded him from returning to his previous independent living arrangement. He demonstrated diffuse, mild weakness throughout the right arm that was variable and seemed to be correlated with his reported pain level, but his most consistent and weakest movement patterns were his grade 4/5 weakness in finger abduction and distal interphalangeal joint flexion. He had reduced pin prick sensation over the volar surface of digits 3–5, palm and forearm of the right arm and hand. He was not able to perform transfers to or from his manual wheelchair due to his level of pain. The magnetic resonance imaging (MRI) revealed a heterogeneously enhancing mass with a maximum diameter of 6.9 cm (Fig. 1) that involved the right deltoid and pectoralis major muscles. His core biopsy demonstrated a STS that was classified as a high grade (III) spindle cell sarcoma.

Oncology recommended treatment for his STS with a course of outpatient neo-adjuvant radiation therapy followed by gross total resection with wide margins. Physiatry pre-operative consult focused heavily on functional prognostication. The patient expressed multiple times that he placed the highest priority on return to his previous modified independent living arrangement and not only survival after his STS treatment course. The patient’s personal values combined with the physiatric assessment informed the pre-surgical planning. In particular a decision was made to take a narrower surgical margin around key muscle group (pectoralis major and deltoids) in order to help maintain the man’s manual wheelchair mobility and his ability to independently transfer himself.

He completed neo-adjuvant radiation therapy in November of 2014 and underwent radical excision of his right shoulder mass with flap closure that December. He began intensive inpatient rehabilitation after he was given clearance to weight bear through his arm about 8 weeks later. Initially he required total assistance for most ADL’s, including transfers, manual wheelchair propulsion, dressing and toileting. Despite the extensive surgery and radiation treatments, the gentleman was able to return to a functional level, approaching his pre-morbid status (modified independence). He was successfully discharged home to live alone in his accessible apartment complex.

## Discussion

This is the first report of STS in a patient with traumatic paraplegia. We could not identify any clear STS risk factors present in this case such as history of local trauma to the right arm or shoulder region. However, we speculate the 30 years of relative upper extremity overload required to meet his functional demands as a person with paraplegia may have contributed. He had suffered from a chronic pain syndrome involving both shoulders and it is possible though unknown whether this chronic shoulder led to any local inflammation. Moreover, some animal studies have suggested a causative link between chronic inflammation and the development of STS [10–12].

The most common location for STS developmentis the area of the extremities (50–60%) and the second most common is the trunk (19%). A frequent clinical presentation for STSs is an enlarging mass of an extremity that gradually grows over months or years. STSs are commonly painless masses, but some patients may develop pain. In our case the patient developed a STS in his deltoid and major pectoralis muscle, but his clinical pattern of hand weakness and paresthesias suggested a secondary compressive injury to his brachial plexus (likely the lower trunk), which is known to commonly present with pain as a symptom [13]. The patient declined electrophysiological testing to better characterize his peripheral nerve injury.

After the appropriate workup and staging of the STS, treatment should be initiated. The mainstay therapy of STS of the extremities is surgery with extensive margins. Combination with pre-operative and/or post-operative radiation is suggested for tumors over 5 cm in size. Limb salvage techniques of the extremity, such as surgery combined with pre- and/or post- radiation therapy, have demonstrated similar survival rates to amputation with superiority in terms of functional outcomes [14, 15]. It is suggested that chemotherapy is added only if high grade, large STSs are present in the extremities [16]. Based on the most up-to-date evidence for the patient’s condition, our medical oncologist suggested a neo-adjuvant course of radiotherapy as an outpatient, followed by a gross total resection of his mass.

The key components for delivering optimal treatment results are the transfer of the patient to a specialized center and a multidisciplinary team approach that combines the expertize from different fields to holistically treat the patient [17]. In such a multidisciplinary team, rehabilitation plays a very important role for the patient’s functional outcome, and its high significance is not limited to the time after treatment, but it also expands to the time before. Pre-rehabilitation is an essential component before the initiation of the therapeutic plan. Pre-rehabilitation for STS is advised in order to assess the premorbid functional level, as well as the medical, social or other environmental issues that could affect the patient’s condition [18]. For the post-treatment care, there are physical therapy protocols that have been developed by some rehabilitation teams, specifically for STS management in order to deliver optimal functional outcomes [19]. Finally, it is essential that any psychosocial issues are addressed by the rehabilitation team in order for the patient to be successfully re-integrated into the community.

In our case the patient presented initially to a specialized center for STS treatment. A multidisciplinary team was formed, comprising a Medical Oncologist, Surgical Oncologist, Radiation Oncologist, and a Physiatrist who sub-specializes in cancer rehabilitation. The patient’s goals were set early on as he clearly expressed that he wanted a treatment that balanced his functional concerns with the curative management of his STS. The pre-rehabilitation assessment focused on functional prognostication and informed the pre-surgical planning for key muscles to be spared, with the narrowest margins possible, in order to maintain key abilities such as transfers and manual wheelchair mobility. The patient regained full functionality of his arm after he underwent intensive post-treatment rehabilitation. Eventually, the patient returned to his previous residence in the community without the need for additional adaptations. Thus, it is evident that pre-rehabilitation is of high importance for such challenging cases, i.e., in SCI patients with STS, informing the post-treatment care plan after upper limb salvage techniques for STS and avoiding the potential detrimental functional effects for the SCI patients.

A STS in the upper extremity of a paraplegic patient poses special challenges for the effective management of the patient. The challenges arise from the fact that the smallest motor function compromise can have a detrimental impact on functional independence, life quality and life expectancy. Studies have shown that due to improvements of the medical management, the age of SCI patients has started to increase and life expectancy has been increasing worldwide [20, 21]. Consequently, diseases that were rarely present in the SCI population will now start to arise as the patients get older, creating both diagnostic and management challenges for Cancer Rehabilitation [22].
